# Stab-Resistant Polymers—Recent Developments in Materials and Structures

**DOI:** 10.3390/polym15040983

**Published:** 2023-02-16

**Authors:** Niklas Panneke, Andrea Ehrmann

**Affiliations:** Faculty of Engineering Sciences and Mathematics, Bielefeld University of Applied Sciences, 33619 Bielefeld, Germany

**Keywords:** body armor, additive manufacturing, functional textiles, sensory textiles, shear-thickening fluid, reinforcement, stab protection, VPAM-KDIW, HOSDB

## Abstract

Stab-resistant garments have been used for centuries, utilizing metals, paper, or polymeric structures, often inspired by natural structures such as scales. Nowadays, stab-resistant vests or vest inserts are used by police and security personnel, but also by bus drivers, ambulance officers, and other people who are empirically often attacked on duty. Since stab protection garments are often heavy and thus uncomfortable and not well accepted, whether in the form of chain-mail or metal inserts in protective vests, researchers are striving to find lightweight, drapable alternatives, often based on polymeric materials. These research attempts have recently focused on textile fabrics, mostly with impregnation by shear-thickening fluids (STFs) or ceramic coatings, as well as on lightweight composites. The first studies on 3D printed polymeric objects with tailored shapes, as well as theoretical investigations of the stab-protective effect of different materials, have been published throughout the last years. Here, we discuss different measurement methods, including dynamic and quasistatic methods, and correlations of stab-resistance with other physical properties, before we give an overview of recent developments of stab-resistant polymers, using different materials/material combinations and structures.

## 1. Introduction

Stab resistant clothing has been used for a long time, mostly by soldiers and nowadays police, but also by other people who feel potentially endangered of being threatened with knives. The first stab-protective armor was made from wood, leather, or horn, followed by metals [1]. While soft body armor was developed to protect the wearer from low-velocity bullets and protection from fast-velocity bullets was also developed after the First World War and improved since [2,3,4], stab resistant garments have been less often investigated. Nevertheless, stab protection is often more important since stabbing accounts for more fatal penetrating injuries than gunshot injuries in many studies [5,6,7,8].

Stab-resistant vests have been shown to significantly reduce the number of fatal injuries due to stabbing [9]. However, stab-resistant body armor is mostly heavy and uncomfortable [10], besides the problem that it does not necessarily protect all relevant body area from knives and spikes, depending on the wearer’s body shape [11]. To motivate police and other people in potentially dangerous situations to wear stab-protection garments, lightweight body armor is necessary, ideally from drapable material with low thermal resistance and high air and water vapor permeability [12,13,14]. Besides, recent body protectors used by the police and soldiers often do not fulfill the requirements defined in various standards for stab protection [15]. This has inspired researchers to investigate textile and other polymer-based stab-resistant garments. These materials and structures are discussed in this review.

The paper is structured as follows: The next section gives a brief overview of different dynamic and quasistatic measurement methods, followed by recent theoretical discussions of body armor and correlations between stab protection and other physical properties found in the scientific literature. As potential polymeric stab-resistance materials, pure textile fabrics are investigated, followed by coated and impregnated fabrics. Going one step further, rigid composites and reinforced polymers are presented, before 3D printed body armor as well as other new polymers and protective structures are discussed.

## 2. Stab Resistance Measurement Methods

Generally, quasi-static and dynamic test methods can be found in the literature. Recent test standard differentiate between gloves, e.g., tested according to the EN388, and other garments. One of the dynamic test methods used to evaluate stab resistance of garments is the German VPAM KDIW 2004, in the newest version from 2011 [16].

**Figure 1:**
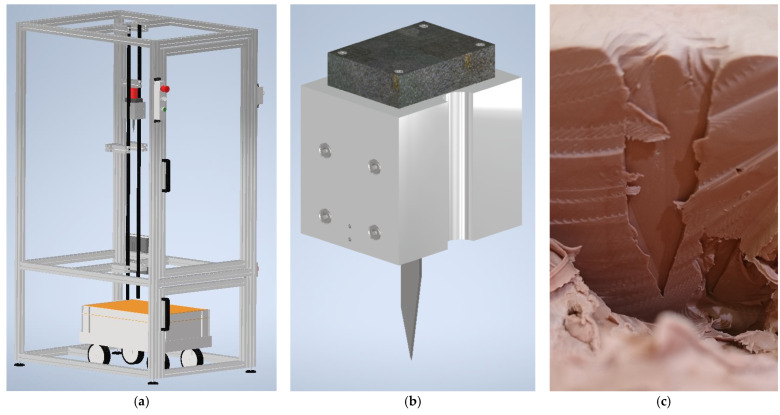
Stab resistance against knives: (**a**) test stand; (**b**) drop mass with standard blade; (**c**) stitch channel during excavation.

This test standard was published by the association of test laboratories for bullet resistant materials and constructions (Vereinigung der Prüfstellen für angriffshemmende Materialien und Konstruktionen, VPAM) and describes testing stab resistance against blade (“Klinge”), spike (“Dorn”), injection cannula (“Injektion”) as well as impact resistance against a block (“Würfel”, combined to the abbreviation KDIW). For stab resistance, the VPAM KDIW defines impact points, strike energies, and angles of incidence which have to be tested to reach different stab resistance classes, such as K1 (knife 1) using a strike energy of 25 J which is reached by letting a blade with a drop mass of 2.5 kg (incl. the test tool) fall from a height of 1.02 m. The test blade and the other test tools are well-defined, as well as the plasticine, the impact locations on the specimen, etc. Figure 1 depicts exemplarily a typical VPAM test stand (Figure 1a) with the standard blade in a drop mass (Figure 1b) and a partly excavated stitch channel (Figure 1c), used to measure the penetration depth. It should be mentioned that stab penetration depths of 20 mm are allowed for blade and spike to pass the test, while injection does not allow any stab penetration.

Another often used standard was published by the British HOSDB (Home Office Scientific Development Branch) [17]. The Home Office Body Armor Standard 2017, based on the HOSDB 2007, is very similar to the aforementioned VPAM KDIW. Instead of the plasticine back, it uses backing foam to test protection against spike and knife. It necessitates two tests with different impact energies for both of the two protection levels, e.g., for level KR1 a maximum penetration depth of 8.0 mm is allowed for an impact energy of 24 J, and a penetration depth of 20 mm for an impact energy of 36 J. Penetration is measured directly by a digital caliper as well as with a synthetic witness paper placed below the stab test sample, allowing the detection the cut length. As opposed to the VPAM KDIW, the spike is not allowed to penetrate the stab resistant material at all. The test sabot in which the knife is embedded contains a damper disk to enhance the realism of the test.

The National Institute of Justice of the USA have published the NIJ standard 0115.00 in 2000 and a draft of the subsequent standard 0115.01 in 2020, defining another test standard for the stab resistance of body armor [18,19]. This standard uses a composite backing material from neoprene sponge, polyethylene foam, and natural rubber, a velocity measuring instrument is located near the test item to measure the velocity of the blade or spike when it reaches the test material. A single-edged blade, similar to the one used in the VPAM, as well as a double-edged blade and a spike are described in the recent draft of the NIJ standard 0115.01. Tests with commercial test threats are performed at energies of 24 J and 36 J, with penetration depths of 7 mm and 20 mm allowed, respectively.

Among the quasi-static test procedures, ASTM F1342 defines a material resistance to puncture [20]. This standard measures the force which is necessary to penetrate a textile fabric, a coated material, or an elastomeric material by a pointed puncture probe. Many reports can be found in the literature about similar tests, using blades, spikes, injection needles, and similar sharp objects.

Especially for gloves, the EN 388 [21] is often used in Europe, in its newest version supplemented by the ISO 13997 which defines mechanical properties of protective clothing [22] and sometimes by an impact resistance measurement according to EN 13594 [23]. The EN388 tests for abrasion, cut, tear and puncture resistance; the performance levels from 1–4 (1–5 in case of cut resistance) are written on the gloves. The ISO 13997 defines a cut test method, sometimes called TDM (tonodynamometer) test, which means that it measures a linear cut with a fixed blade through the examined material, while the coupe test applied in EN 388 for the cut resistance uses a rotating knife. For gloves used in situations where higher impact or vibration hazards may occur, the EN 13594 measures the impact protection. Both of these values can be given with letters behind the aforementioned numbers on a glove, where A-F define the cut resistance level according to ISO 13997, while an additional P informs that the impact protection test according to EN 13594 was passed.

While these tests are standardized, several research groups show experiments according to modified test parameters, such as different blade shapes, different impact energies, or quasi-static tests with different sample holders than specified in the respective standards, leading to the necessity to carefully interpret the results achieved with different test equipment [24]. Generally, quasi-static tests, as also mentioned in the NIJ Standard 0115.00, are performed on universal test machines, with usually the upper clamp holding a standardized blade, while the lower clamp is exchanged by either a sample holder in which the investigated sample is clamped, or by a backing of different foams and sponges as in the dynamic tests. In this way, the load-displacement curve during the quasi-static stabbing process is recorded, before the test is stopped at a defined displacement.

Finally, it should be mentioned that the often used maximum penetration depth of 20 mm is related to the fact that this value is the median distance of vital organs from the skin surface, while the minimum distance was around 10 mm [25], which explains the other often chosen value of 7 mm.

## 3. Modeling and Correlations with Other Physical Parameters

While many research groups report on experimental investigations of stab resistant garments, few studies can be found in the literature describing the mechanism of penetration of a blade, a spike, or a needle through body armor or potential correlations with other physical parameters, such as thickness, density, elastic modulus, or other properties of protective materials. This is the opposite to body armor against hand-gun bullets, where different parameters play the main role, and which has been investigated intensively [26].

The forces necessary to penetrate the human skin were experimentally found to be in the range of 5–30 N [27], with clothes having different impact on this value, depending on the shape and sharpness of the chosen blade [28]. Horsfall reviewed existing studies regarding the shape and sharpness of the blade and concluded that energy absorption was performed by friction, bending of the cut material, and finally fracture and other mechanisms at the crack tip, with different amounts of friction and bending being reported, depending on the blade geometry [29]. He also mentioned the often large difference between impact energies in dynamic tests and quasi-static energies, leading to identical penetration depths, showing that dynamic and quasi-static test procedures can not always be compared reliably.

**Figure 2:**
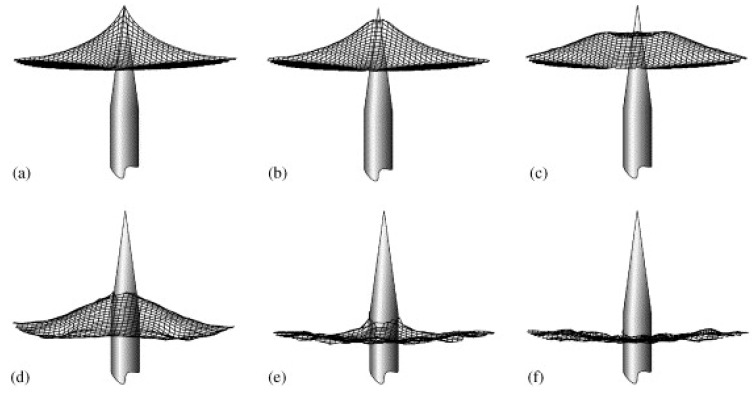
Simulated morphologies of deformation for a needle going through a single ply of plain weave Kevlar fabric. (**a**–**f**) define increasing displacement. Reprinted from [30], Copyright (2006), with permission from Elsevier.

An early model of the stab resistance of fibrous systems was suggested by Termonia, taking into account single-ply and also multi-ply fabric systems [30]. He modeled a woven fabric in which the yarns can slip over each other at the crossing positions, held by circular clamps, into which a needle is inserted with constant velocity. Depending on the deformation morphology, as depicted in Figure 2, he calculated different forces for the needle penetrating through a Kevlar plain weave fabric, where the largest force was reached (Figure 2a) directly before the fabric is punctured (Figure 2b), while further displacement of the yarns around the needle let the force increase again (Figure 2d,e), before the cylindrical part of the needle with constant diameter is reached, and force as well as fabric deflection are decreased again (Figure 2f).

For weft-knitted aramid fabrics in a flexible Surlyn resin matrix, Liu et al. found that optimization of the hot-pressing temperature, the pressure and holding time, as well as of the resin content, could improve the stab resistance properties of the prepared composites, which they attributed to a combination of shear force, tensile fracture, friction, and deformation of yarns and fabric as potential energy dissipation mechanisms [31].

A numerical model of Barnat et al. for an aramid fabric stack of 35 layers was compared with the optical investigation of the stabbing process, using a high-speed camera with 5000 frames/s [32]. The researchers used the movies to plot the knife velocity and acceleration and showed that the numerically calculated and the measured displacement vs. time were in good agreement. They described subsequent tearing of the roving bundles after first moving them away by the knife, and their calculations showed an increasing contact force between blade and fabrics with increasing penetration into the stack of fabrics.

**Figure 3:**
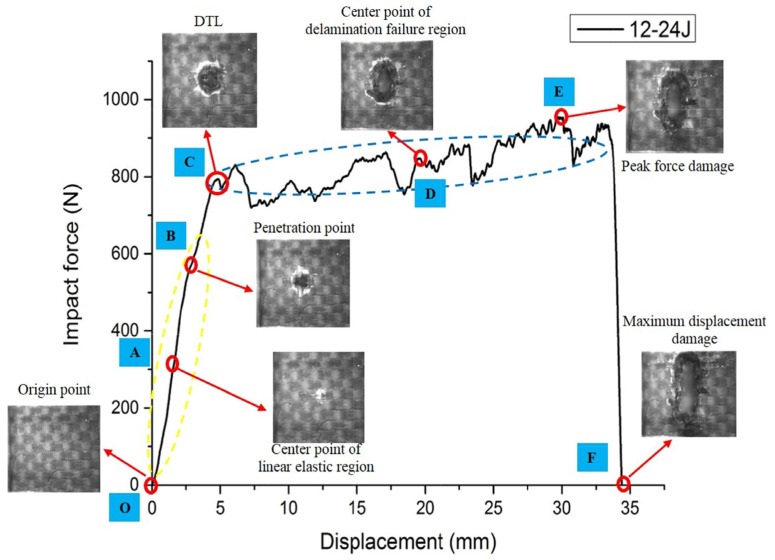
Measured characteristic points and local failure modes of 12-layer composite on the impact force-displacement curve at 24 J impact energy, combined with high-speed camera images of characteristic points. DTL means delamination threshold load. Reprinted from [33], Copyright (2022), with permission from Elsevier.

A high-speed camera was also used by Du et al. who recently used this technique to monitor the damage morphology on the material back during puncture [33]. Opposite to Barnat et al., they worked with quasi-static and dynamic puncture tests on a carbon fiber reinforced polymer instead of pure fabrics. They characterized the local failure modes of a laminate with 12 layers of carbon fiber fabrics, as depicted in Figure 3, as a nearly linearly increasing impact force until the delamination threshold load (DTL) was reached, followed by increasing force with proceeding delamination failure, until the peak force damage is reached shortly before the maximum displacement, characterized by the impact force vanishing. In theoretical calculations and measurements, they showed a disproportionately high increase in the absorbed impact energy with increasing material thickness.

While the aforementioned thickness dependence of the stab resistance of a fabric or composite seems obvious, Guo et al. studied other parameters of polymers used for stab resistance [34]. Combining experiments and numeral simulations, they found the largest effect caused by the shear strength and the surface hardness of the examined materials, making polycarbonate (PC) ideal for stab resistance and polyethylene (PE) the worst.

As these examples show, the general process of stabbing into a material is generally mostly understood, with many authors subdividing it into the three phases of cavitation, penetration, and perforation [34], partly using other nomenclature. Nevertheless, in a real textile fabric, polymer plate, composite, or similar polymeric object, many interactions occur, hampering the simple calculation of the stab resistance by measuring some other parameters. The following sections thus give an overview of recent approaches to improve the stab resistance of polymeric objects, including new polymers, new shapes, new techniques such as additive manufacturing (3D printing), and more and more improved textile fabrics with different coatings and impregnations.

## 4. New Shapes and Polymers

One possible approach to improving body armor for stab resistance is based on developing new shapes or new polymers, e.g., inspired by nature. Such bio-inspired structures can be found in many areas, such as energy absorption [35], impact resistance [36], or biological armor design [37]. While 3D printing (described in Section 5) offers the largest degree of freedom and is thus often used to prepare such bio-inspired stab-resistant structures, there are nevertheless a few other approaches to preparing bio-inspired armor without additive manufacturing.

**Figure 4:**
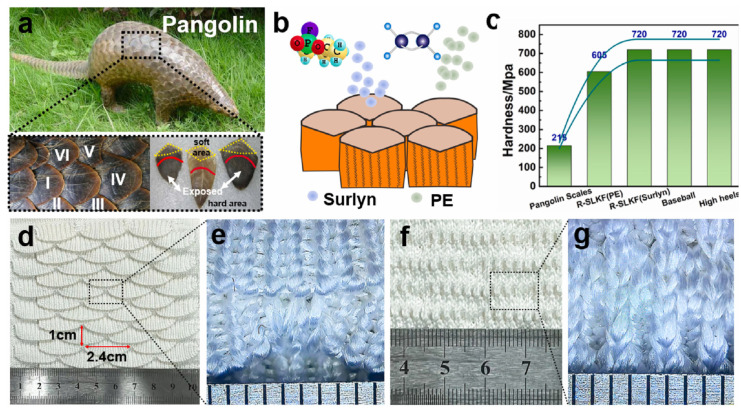
(**a**) Pangolin and its scales. (**b**) Preparation diagram of reinforced scale-like knitted fabric (R-SLKF). (**c**) Hardness of R-SLKF and comparison with other materials. (**d**) Optical image on the front of scale-like knitted fabric (SLKF) and (**e**) enlarged view. (**f**) Optical image on the back of SLKF and (**g**) enlarged view. Reprinted from [38], Copyright (2022), with permission from Elsevier.

Liu et al. prepared a scale-like structure composite from a knitted fabric reinforced with resin, inspired by pangolin scales [38]. Figure 4 depicts the pangolin with its scales (Figure 4a), the idea of reinforcing the double-layer scale-like knitted fabric with Surlyn and polyethylene (PE) resins only at the scales, while the lower fabric parts stay untreated, the measured hardness values compared to real pangolin scales, and an untreated scale-like knitted fabric from front and back (Figure 4d–g). By restricting the resin to the movable scale structure, the authors showed that the composite stays relatively flexible, while the stab-resistance could significantly be increased by infusing the upper fabric part with resin.

A warp-knitted scale structure was prepared in the same lab, in this case using epoxy resin with SiC particles to impregnate the scales [39]. They reported an increase in the stab resistance due to the addition of SiC particles, but also mention that the knitted material (here polyester and Spandex) should be changed to a technical yarn, and washing of the composite structure should be tested.

Besides these ideas to improve stab resistance by sophisticated structures, other research groups concentrated on developing new polymers or blends to enable the production of better body armor, e.g., by making it flexible. Yong prepared flexible composites from rubber wood fiber mats in a rubber matrix including silica filler to improve the fiber-matrix adhesion [40]. He reported good elastomeric behavior at a high fiber loading of about 33 wt% and medium stab-resistance level, tested according to NIJ 0115.00, passing the tests for knives with one and two cutting edges at threat levels 1 (impact energy 24 J) and 2 (33 J), while level 3 (43 J) was not reached. The test with the spike was only passed under an angle of incidence of 0°, not of 45°, at level 1, a finding which is often reported since pointed weapons are generally harder to stop with textile-based objects.

A non-textile approach to building flexible, often even stretchable stab-resistant objects is based on hydrogels. Nakahata et al. prepared a hydrogel containing β-cyclodextrin-acrylamide and adamantane-acrylamide which was not broken by strong compression after crosslinking, could be stretched over a pencil or a cutter-blade, and showed self-healing properties [41]. Similarly, Tan et al. used free-radical copolymerization of acrylamide and adamantane-2-isocyanatoethyl acrylate-β-cyclodextrin to prepare a supramolecular hydrogel with high notch resistance, stab resistance tested by sharp scissors, and self-healing properties [42]. Using a poly(acrylamide)/poly(ethylene oxide)/LiCl hydrogel, Li et al. prepared strain sensors which also showed a high stab-resistance and self-healing properties [43].

It must be mentioned that these pure hydrogels are not tested according to standards for body armor, but are used in other stab-resistance applications, where much smaller impact energies or quasi-static forces are expected. This is often different for the 3D printed structures discussed in the next section.

## 5. 3D Printed Polymer-Based Body Armor

3D printing, or additive manufacturing, summarizes several different technologies, typically based on material extrusion, such as the most often fused deposition modeling (FDM) technique, on photopolymerization of a resin, such as stereolithography (SLA), or on melting defined positions inside a powder bed, such as selective laser sintering (SLS), while a few techniques have other principles, e.g., combining powder with a glue [44].

**Figure 5:**
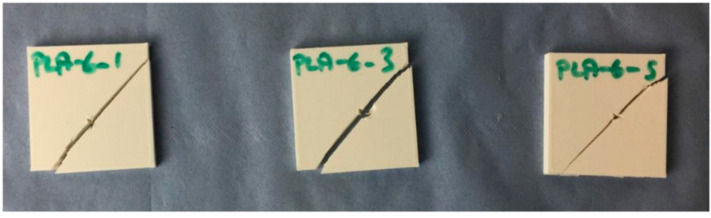
Fractured PLA specimens after stab test. Reprinted from [45], originally published under a CC-BY-NC license.

Using FDM, Cicek et al. printed square specimens of lateral dimensions 40 mm–80 mm with thickness from 6–10 mm from acrylonitrile butadiene styrene (ABS), poly(lactic acid) (PLA), and other materials with an infill of 100% [45]. They tested the samples according to the HOSDB level KR1-E1, meaning an impact energy of 24 J. While they found suitable mean penetration depths around 8 mm for ABS, PLA was found to fracture in some tests, as shown in Figure 5. The fracture lines are oriented along the 45° direction, i.e., along one of the printing orientations (in FDM, the molten polymer is typically placed under ±45° in subsequent layers). Besides, the authors report that polycarbonate (PC) and tough PLA (TPLA) were found most suitable for stab resistance, necessitating structures of only 5 mm thickness. Similar results were reported by Maidin et al. who used FDM printing of ABS and PC-ABS samples which were tested according to HOSDB KR1-E1 with an impact energy of 24 J, and fractured samples were found in many cases, where PC-ABS showed higher stab resistance than ABS, and an optimum thickness of 8.0 mm was defined [46].

Sample fracture was also reported by Jiang et al. who used SLS to produce plates with a pyramid structure with different tilt angles from polyamide (PA) [47]. They found plate thicknesses around 7.5 mm to be sufficient to fully block a knife which impinged with an impact energy of 24 J for tilt angles from 20–30°, fulfilling the Chinese GA 68-2008 National Standard, while for a tilt angle of 35°, even a thickness of 8 mm was not sufficient. The same structures were investigated by Gong et al. who tested different PA materials, partly including glass fiber, with very similar results [48]. Including PA/carbon fiber, the same structures were tested again, where the PA/carbon fiber specimens needed only a thickness of 6.5 mm to show sufficient stab resistance [49].

**Figure 6:**
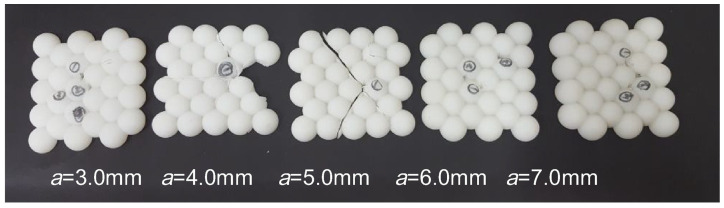
Stab resistant test results of samples with increasingly steeper egg-shell cores (from left to right). Reprinted from [50], Copyright (2018), with permission from Elsevier.

Another shape was investigated by He et al. who used laser sintering of PA to produce egg-shell-like scale structures [50]. In their experiments, according to GA 68-2008 National Standard with an impact energy of 24 J, fractures were sometimes found along the weaker areas between the egg-shell parts, as visible in Figure 6. A larger body armor part was built by partly overlapping sections with such egg-shell structures in different ways, leading to sufficient stab protection of the optimized design which had an areal density of 7.3 kg/m², which is more than one-third less than common stab resistance body armor vests.

A special FDM apparatus, the Markforged Mark Two, was used by Sitotaw et al. to produce nylon/aramid specimens with well-defined aramid filament orientations in each layer [51]. Stab tests were performed with an impact energy of 25 J according to class K1 of the VPAM-KDIW. While pure nylon samples of a thickness of 3 mm always showed penetration larger than 70 mm and thus clearly failed, samples of identical thickness with unidirectional fiber orientation interestingly also failed, independent from the orientation of the impacting knife with respect to the fiber orientation. Combining fiber orientations of 0°/30°/60°/90°/120°/150° as well as 0°/45°/90°/135°, however, resulted in mean penetration depths of 17 mm and 15 mm, respectively, showing the severe influence of fiber oriented in such a composite.

A similar printer, the MarkOne by Markforged, was used to prepare different scale structures as well as fiber-reinforced polymer samples [52]. Ahrendt et al. found that 4 mm thick 3D printed fiber-reinforced polymers specimens with different fiber orientations in subsequent layers fulfilled the stab protection level K2 of the VPAM-KDIW, meaning that the penetration depth for an impact energy of 25 J was smaller than 5 mm on average and 10 mm maximum.

Generally, 3D printing with its diverse technologies offers many possibilities to prepare interesting shapes for stab-resistant body armor; however, the best results are reached with composites that contain fibers or filaments in defined orientations. This is why the next section gives an overview of reinforced polymers and composites which are prepared with traditional techniques.

## 6. Reinforced Polymers and Composites for Stab Resistance

Among the textile fibers, yarns, woven fabrics and nonwovens used in fiber-reinforced polymers as well as in composites, aramid belongs to the most often reported materials. Aramid, or more exactly *p*-aramid (poly(*p*-phenylene terephthalamide), PPTA), is an aromatic polyamide showing a very high crystallinity, which together with strong intermolecular hydrogen bonds makes the material very strong and thermally stable [53]. Kim and Nam investigated composites from *p*-aramid fabrics in thermoplastic low-density poly(ethylene) (LDPE) and epoxy resin with thicknesses of 11–16 mm [54]. Applying quasi-static and drop-tower tests according to NIJ standard 0115.00, epoxy resin of 43 wt% add-on to the woven aramid fabric was proven to optimally stop the impinging knife in the drop tower tests.

Stojanovic et al. combined multiaxial and woven *p*-aramid fabrics, impregnated with polyurethane (PU), and laminated them with thermoplastic PU from one side [55]. Afterwards, they were impregnated with silica nanoparticles in a poly(vinyl butyral) (PVB) matrix. Stab resistance was tested with a quasi-static procedure according to NIJ standard 0115.00. The authors reported improved mechanical and stab-resistance properties upon addition of amino-modified SiO_2_.

A matrix of PVB with small fractions of inorganic fullerene-like WS_2_ nanoparticles and WS_2_ nanotubes was chosen to produce composites with woven and cross-plied Kevlar [56]. Simic et al. found in quasi-static tests according to NIJ 0115.00 a significant increase in the absorbed energy of a knife stab and of the deformation depth for the samples with these nano-reinforements.

Stab and puncture resistance of a woven Kevlar (brand name of a *p*-aramid) fabric were investigated by Zhao et al. who concentrated on the impact of a sizing agent on the composite’s mechanical properties [57]. The authors prepared a sizing agent from water-borne epoxy resin, poly(acrylamide) and fatty alcohol polyoxyethylene phosphate potassium salt in which they immersed the fabrics. The resulting composites were investigated by quasi-static tests according to ASTM F1342-05, applying a spike, a knife blade and a bursting impact head. Thy found an optimum sizing rate of 10 wt% for puncture and stab resistance as well as burst strength, with an increase by a factor of approx. 3–7 as compared to the pure fabrics which they attributed to the immobilized fiber bundles in the impregnated fabrics.

**Figure 7:**
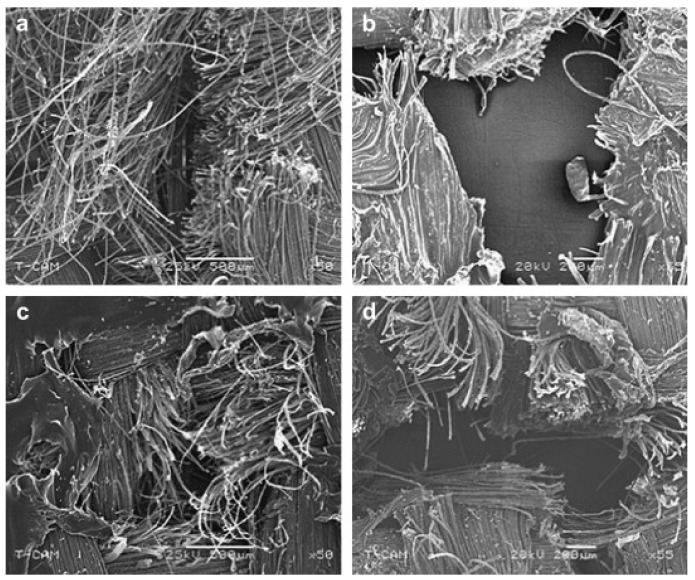
Scanning electron micrographs of (**a**) pure Kevlar, (**b**) PE composite, (**c**) Surlyn/PE composite, and (**d**) Surlyn composite after dynamic stab testing. Reprinted from [58], Copyright (2009), with permission from Elsevier.

Polyethylene (PE), Surlyn^®^ (poly(ethylene-*co*-methacrylic acid) and Surlyn/PE bilayer films were used to prepare composites with Kevlar in different thicknesses between 2.67 mm and 7.95 mm [58]. Mayo Jr. et al. performed quasi-static and dynamic tests according to NIJ standard 0115.00 to investigate stab and puncture resistance of these specimens. For the quasi-static and dynamic knife tests, all samples showed damage zones with fiber fracture, as depicted in Figure 7, while the overall stab-resistance was best for Surlyn impregnation and significantly lowest for pure aramid.

Besides aramid, other technical fibers or filaments have been used in stab-resistant composites. Li et al. used ultrahigh molecular weight polyethylene (UHMWPE) impregnated with different thermoplastic films and found especially polyethylene terephthalate (PET) and polypropylene (PP) films better suited than polyethylene (PE) [59]. For stab energies of 24 J, the penetration depth of samples with an areal weight of 8 kg/m² with PET and PP films was below 20 mm. Firouzi et al. suggested nylon 6,6 and nylon 6,12 coatings on UHMWPE fabrics to improve their stab resistance in dynamic and quasi-static tests [60].

**Figure 8:**
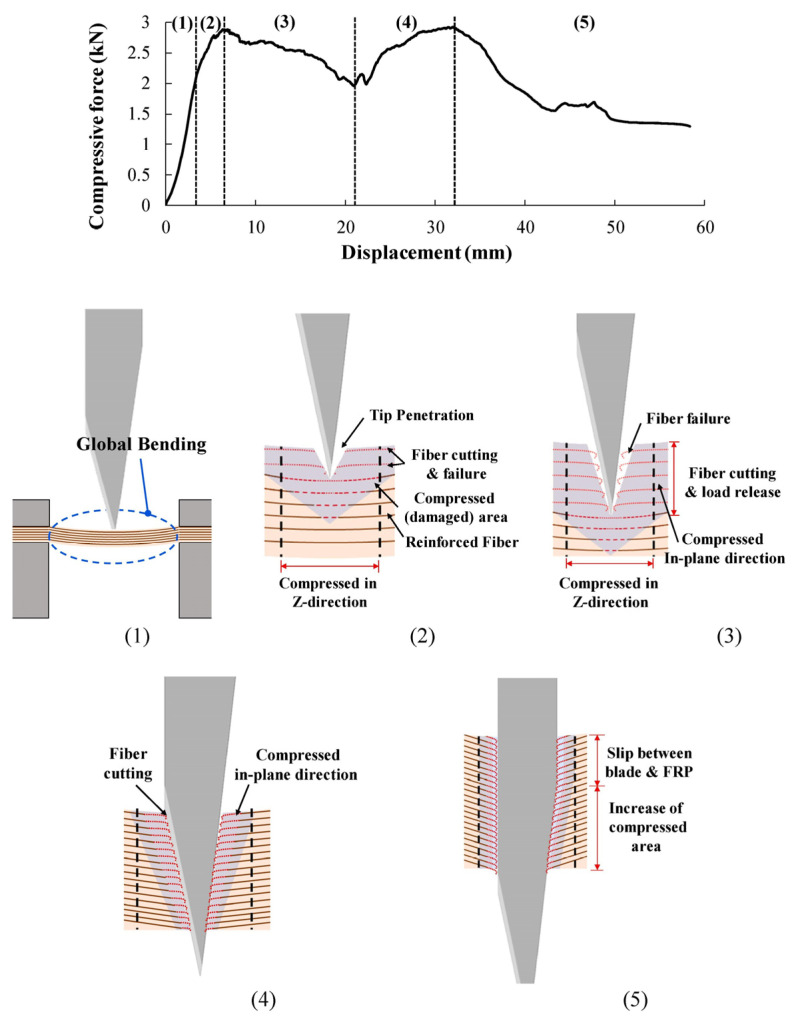
Blade penetration and stab resistance mechanism of the fiber-reinforced polymer composites. Numbers (1)–(5) correspond to the positions marked in the upper diagram. Reprinted from [61], Copyright (2020), with permission from Elsevier.

Carbon, E-glass, and *p*-aramid fibers were compared by Cheon et al. who prepared fiber-reinforced polymer composites with an epoxy matrix and 6–24 fabric layers and tested their stab-resistance according to the NIJ standard with a drop tower as well as in a quasi-static test [61]. Besides these samples, hybrid composites from carbon/aramid and carbon/glass were investigated with 8 layers per material in different orders. Depending on the material, each layer had a thickness of 0.22 mm to 0.26 mm and an areal weight of 313 g/m² to 426 g/m². The authors found a clear dependence of the penetration depth on the thickness and areal weight, i.e., on the number of layers for all reinforcement materials, as expected. From the quasi-static tests, they described the blade penetration and stab resistance mechanism of the samples, as depicted in Figure 8. Besides, they found the carbon fiber reinforced polymer composite to show optimum stab resistance, with a thickness of 2.6 mm being sufficient to reach level 1 of the NIJ standard (<7 mm penetration depth), while the glass composites needed a thickness of 3.2 mm and the aramid composite needed a thickness of 3.9 mm, making carbon an interesting material for this application.

An interesting material combination was suggested by Chuang et al. who prepared PET/PET fiber/matrix composites with woven carbon, aramid, and basalt fabrics [62]. They combined recycled high-strength polyester with low-melting point polyester, needle-bonded them to form multilayer fabrics on both sides of a woven fabric, and used hot-pressing to form a composite bonded by the low-melting point polyester. Quasi-static puncture tests were performed according to ASTM F1342, showing highest puncture resistance for the highest amounts of recycled high strength PET fibers, while no significant differences were visible comparing basalt, carbon, and Kevlar woven fabrics. It should be mentioned that these experiments, although declared as stab tests, are actually puncture tests and thus only partly comparable with real stab tests.

Khuyen et al. avoided technical fibers and investigated the stab-resistance of composites with linen and silk plain weave fabrics in water-based PU, urea formaldehyde (UF) and poly(vinyl alcohol) (PVA) matrices [63]. The highest tensile strength was found for linen/UF; manual (and thus highly subjective) stabbing tests were performed with an undefined blade, where 20 layers of hard silk/UF, resulting in a thickness of 7.9 mm, showed sufficient results. Unfortunately, the different blade and the manual stabbing make a comparison with other experiments highly unreliable.

**Figure 9:**
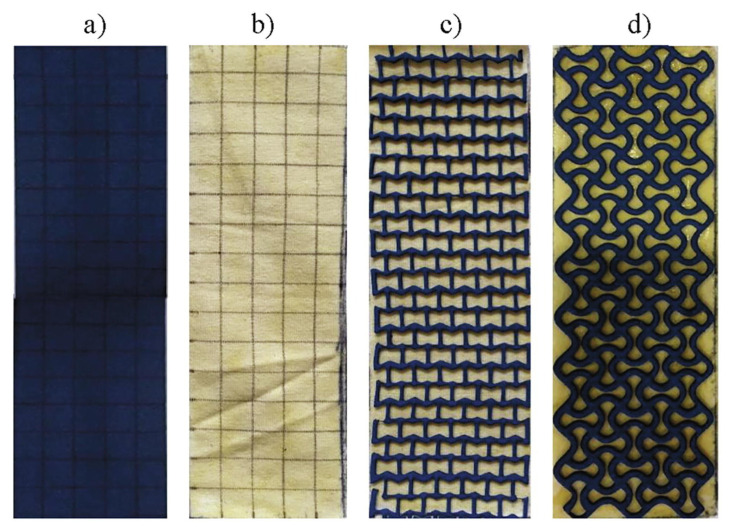
Analyzed specimens: (**a**) EVA foam, (**b**) fabric (with an adhesive), (**c**) re-entrant auxetic laminate, and (**d**) chiral auxetic laminate. Reprinted from [66], Copyright (2020), with permission from Elsevier.

Another way to optimize stab-resistant body armor is to concentrate on the structures in which common materials are used, as described in the previous sections. For this purpose, composites can contain auxetic structures, i.e., structures with negative Poisson’s ratio which give them special mechanical properties [64]. Xu et al. used auxetic warp-knitted spacer fabrics with two-component silicone rubbers with different filling rates and showed that a larger auxetic effect resulted in less damage which they attributed to denser face layer structures and more deformable units of fabrics with larger negative Poisson’s ratio [65]. Novak et al. combined auxetic and non-auxetic layers, laminating non-auxetic cotton/elastane weft-knitted fabrics with a structured ethylene-vinyl acetate (EVA) foam with two different auxetic cellular structures (Figure 9), bonded with rubber-based adhesive [66]. While tensile force-displacement tests showed partly auxetic behavior of the laminates, stab resistance was here only suggested as a potential application of such structures and not measured. For a re-entrant auxetic weft-knitted aramid fabric without lamination, however, quasi-static tests showed a significantly higher energy absorption by the auxetic fabrics, as compared to plain weft-knitted fabrics [67]. In spite of these interesting results, composites with auxetic properties are only scarcely investigated with respect to their stab-resistant properties.

Generally, different polymers can be used to glue the fibers or filaments of a textile fabric together and thus increase the fiber/fiber friction, leading to improved stab resistance [68,69,70]. On the other hand, many textile fabrics with a coating of silica or other ceramic materials are reported as lightweight stab resistance materials. The next section gives an overview of such coated textile fabrics.

## 7. Textile Fabrics with Ceramic Coatings

**Figure 10:**
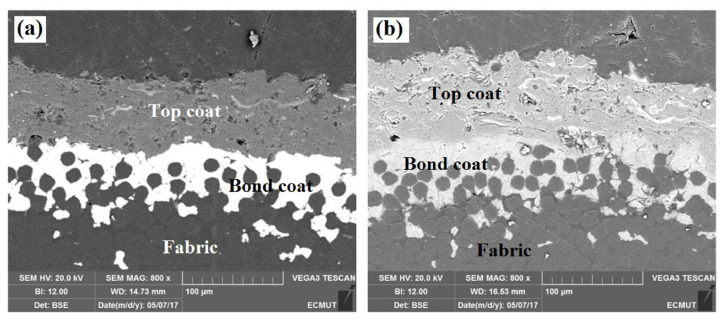
SEM image of the cross-section of aramid fabric coated with (**a**) Cu/ Al_2_O_3_–13%TiO_2_ and (**b**) Al/ Al_2_O_3_–13%TiO_2_. Reprinted from [73], Copyright (2020), with permission from Elsevier.

Ceramic coatings can be used to increase the fiber/fiber friction as well as the hardness and wear resistance, in this way damaging the penetrating blade and thus reducing its ability to cut subsequent fibers [71,72]. Manaee et al. suggested an Al_2_O_3_/TiO_2_ plasma-sprayed ceramic coating to increase the stab resistance of aramid fabrics [73]. They applied copper and aluminum metal powder as bond coat material and Al_2_O_3_–13%TiO_2_ as the top coat, as visible in Figure 10. While the tensile strength remained unchanged by these coatings, quasi-static stab tests showed a severely improved penetration work by Al_2_O_3_–13%TiO_2_ with either metallic bond coat, in both cases approx. five times the value of the uncoated fabric, which the authors attributed to fixing the aramid fabric and increasing the fabric hardness, or in other words improving abrasion and friction by the coatings.

Boron carbide (B_4_C) coatings on aramid/ballistic nylon were investigated by Nayak et al. who showed in quasi-static tests according to NIJ 0115.00 a significant increase in the stab resistance, as compared to the pure textile fabric [14]. Similarly, a boron carbide coating in epoxy resin on aramid fabrics reached an approximately five times puncture load in quasi-static puncture tests, as compared to the uncoated textiles [74]. Applied on UHMWPE fabrics, B_4_C could also improve the stab resistance approx. by a factor of five [75].

Besides boron carbide, silicon carbide was investigated as a coating on stab-resistant plain-weave thermoset-aramid composites [76]. Wei et al. sprayed SiC powder in water with dispersing agents and PVA as a binder on the fabrics and finally impregnated them with vinyl ester resin. Dynamic stab tests according to NIJ standard 0115.00 showed a clear correlation of decreasing penetration depth with increasing SiC concentration up to a concentration of 20 wt%.

Applying SiO_2_ coatings on woven aramid fabrics, Javaid et al. showed more than doubled knife penetration resistance as compared to the pure fabrics, which they attributed to an increase in the yarn-yarn friction [77]. A PU/*p*-aramid multiaxial fabric was coated by PVB with SiO_2_ nanoparticles and carbon nanotubes, resulting in improved wear resistance and 35% higher absorbed energy as compared to the PVB coated samples [78]. Similarly, Kanesalingam et al. observed an increase in quasi-static stab and puncture resistance for silica coatings on Kevlar/wool and Kevlar/wool-nylon fabrics, but at the same time an increase in fabric stiffness, which reduces ergonomics and wearability [79].

It should be mentioned that ceramic aerogels have been suggested as temperature sensor materials for firefighters, so that a combination of improved stab resistance and sensory properties may also been taken into account, depending on the specific application of a stab-resistant garment [80,81,82].

Besides such ceramic coatings, an interesting approach which is heavily investigated in recent years is based on coatings with shear thickening fluids (STFs), which will be discussed in the next section.

## 8. Textile Fabrics with Shear-Thickening Fluid

Shear-thickening fluids are non-Newtonian fluids which show an increase in viscosity with increasing stress or shear rate [83,84]. Such STFs are prepared by dispersing concentrated colloidal suspensions of solid particles in a liquid, where the transition from low to very high viscosity is based on forming transient aggregates upon shear [85,86]. They are thus expected to not significantly impact the wearing comfort of an STF-coated stab-resistant garment, while stiffening in case of an impact and thus offering higher stab protection than uncoated textile fabrics [87]. This promising material class has been investigated deeply in recent years.

**Figure 11:**
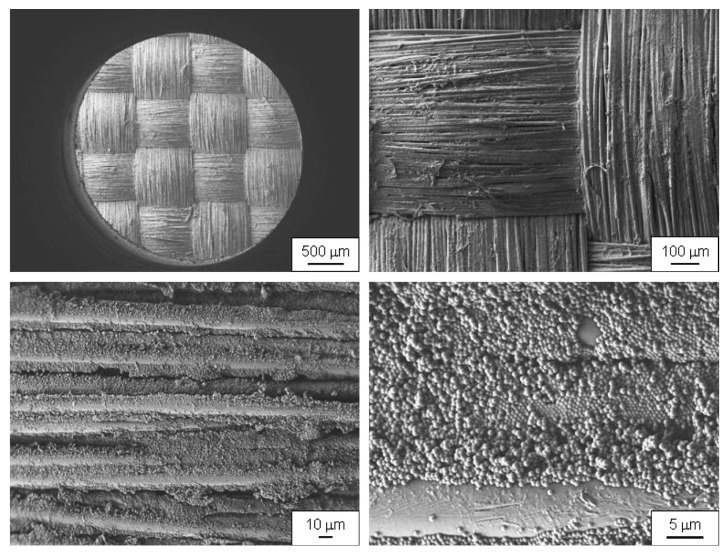
SEM images of undamaged Kevlar coated with STFs in different magnifications. Reprinted from [88], Copyright (2007), with permission from Elsevier.

Decker et al., e.g., prepared STFs by dispersing colloidal silica particles with average diameter 450 nm in poly(ethylene glycol) (PEG) with molecular weight 200 Dalton and coated plain-weave Kevlar and nylon fabrics with them by diluting the STFs in ethanol and soaking the fabrics in this fluid [88]. The resulting coated fabrics are depicted in Figure 11, where PEG is mostly evaporated in the vacuum of the scanning electron microscope (SEM) chamber. Drop tower tests according to NIJ standard 0115.00 with knife and spike showed that a 12-layer STF/Kevlar specimen provided better protection than a 15-layer pure Kevlar sample, while both had similar areal weights, but the STF coating increased neither thickness nor bending rigidity, so that an additional STF coating could lead to preparing thinner, more flexible body armor. In quasi-static tests, STFs significantly increased the cut resistance of the Kevlar sample. Similar results were found for STF-coated nylon fabrics, which in general showed less stab resistance than Kevlar samples.

Kang et al. prepared SFT by dispersing fumed silica particles in methanol and blending this dispersion with medium fluid ethylene glycol [89]. Kevlar plain weave fabrics were immersed in this STF dispersion, squeezed, and dried. While stress-strain curves of untreated and coated Kevlar fabrics, bending angle and thickness were similar, quasi-static stab tests showed significantly higher loads and less damage for the coated fabric. Similar experiments, coating aramid or UHMWPE with varying silica/PEG STFs with a typical amount of 35–55% silica, with comparable results were also reported by other research groups [90,91,92,93,94,95]. The STF preparation process was slightly varied in some studies, e.g., by adding a silane coupling agent to improve silica/PEG bonding and thus form siloxane (Si-O-Si) bonds [96], comparing different silica particle shapes [97] and particle sizes [98], or varying the impregnation pressure to increase the STF loading of the fabrics [99]. Asija et al. used poly(propylene glycol) (PPG) instead of PEG as a base material for STF preparation [100]. Different ionic liquids instead of PEG were tested by Qin et al. who prepared STFs from silica microspheres in these ionic liquids by ultrasonication [101]. They found an optimum stab resistance for an STF loading of 35 wt% on the tested Kevlar fabrics and measured that the yarn-yarn friction was increased by more than one order of magnitude by the addition of the STF, as compared to the pure aramid fabric.

**Figure 12:**
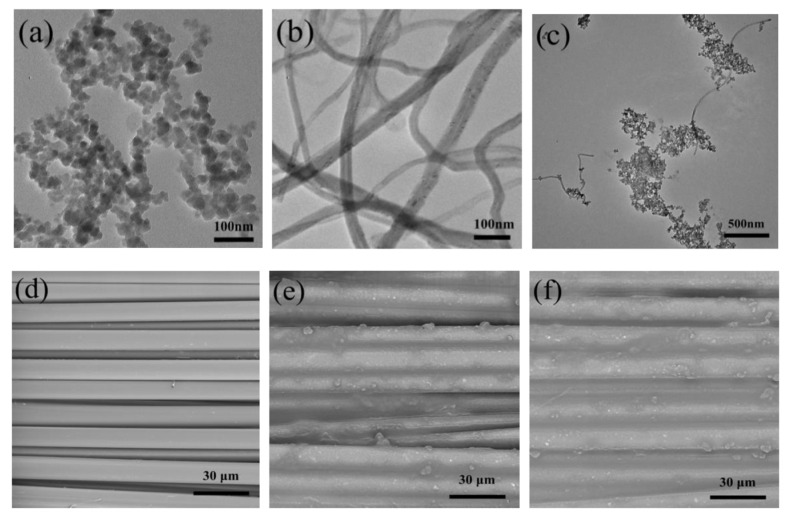
TEM images of (**a**) fumed silica nanoparticles, (**b**) O_2_-plasma treatment MWNTs and (**c**) fumed silica nanoparticles and M-MWNTs, SEM images of (**d**) neat Kevlar fabric, (**e**) STF/Kevlar fabric, and (**f**) M-MWNT/STF/Kevlar fabric. Reprinted from [102], Copyright (2018) by the authors, originally published under a CC-BY license.

To further improve STF coatings, several studies combined them with additional nanoparticles, carbon nanotubes etc. Li et al. suggested STFs combined with multi-walled carbon nanotubes (CNTs) for improved stab resistance of Kevlar plain-weave fabrics, as depicted in Figure 12 [102]. In quasi-static stab tests, a significant increase in the peak force for STF-coated Kevlar fabrics was found, while CNT/STF coatings further increased the peak force values. The authors attributed this result to increased rigidity and decreased yarn mobility upon the coating.

Carbon nanotubes were not only shown to increase the stab resistance properties of STF/Kevlar specimens [103], but were also sometimes discussed in terms of their conductivity, enabling the use of such fabrics as body movement sensors, etc. [104,105].

Different nanoparticles were also added to STF-coatings on stab-resistant fabrics. Gürgen and Kushan as well as Gürgen and Yildiz added SiC particles of different sizes, leading to an increased viscosity profile of the STFs and thus higher energy dissipation, as well as to a reduced penetration depth in drop-tower tests with spike and knife [106,107,108].

Combinations of STF and polymer coatings were suggested, e.g., by Zhang et al. who combined nonwovens from recycled Kevlar and nylon fibers on both sides of an aramid fabric by needle-punching, impregnated them by STF, and finally coated them with thermoplastic polyurethane (TPU) [109]. In dynamic and quasi-static tests according to ASTM F1342-05 with spike and knife, they found an increase in the stab resistance due to STF coating as well as due to the TPU coating, with the highest stab resistance reached by combining both treatments. Combining STF with a TPU coating including fumed silica was suggested by the same group and led to a significantly improved quasi-static stab and puncture performance, with the optimum stab resistance reached for a coating with 3% fumed silica [110].

As these examples show, a large part of recent research in stab-resistance reached with polymeric materials is based on finding new, optimized coatings for aramid and other high-tenacity fabrics. However, investigations of the fabrics themselves are also important to further improve the textile components in composites and coated fabrics. The next section gives an overview of recent studies of pure stab-resistant textiles.

## 9. Pure Textile Fabrics for Stab Resistance

Pure textile fabrics receive their stab-resistant properties partly from the fiber, filament or yarn properties, i.e., from the fiber/filament material and titer as well as the production of the yarn. Mayo Jr. and Wetzel compared different technical fibers, such as different *p*-aramids and UHMWPEs, carbon, and S-glass, and pressed an industrial cutting blade laterally on the single fibers to investigate the angle-dependent failure [111]. They found similar cut resistance levels for all fiber types, with generally higher average cut resistance of the inorganic fibers due to their hardness.

Tian et al. suggested using Kevlar- and UHMWPE-covered yarns and reported Kevlar fiber wrapping around a core fiber leading to enhanced cut resistance, as compared to pure Kevlar or pure UHMWPE yarns [112]. On the other hand, the cut resistance decreased with increasing twists of the covered yarns. The authors thus suggested testing such covered yarns for stab-resistant garments.

Diverse technologies exist to prepare fabrics from these and other yarns or fibers. Needle-punching is an often used method to create nonwovens. However, needle-punched composites are usually applied in the form of composites with epoxy resin, thermal bonding of low-melting point fibers, or similar matrices, when they are to be used in stab resistance applications [113,114], and investigations of pure nonwovens for this purpose are hard to find.

Knitted fabrics are also used only scarcely for stab-resistance applications, which can be explained by their elasticity. Sun et al. nevertheless performed quasi-static stab resistance tests on auxetic weft-knitted Kevlar fabrics and found a higher stab resistance than on plain weft-knitted Kevlar fabrics, where the break-points in the auxetic fabric could be significantly reduced by alternating face and reverse loops [66].

Liu et al. combined UHMWPE with a polyamide/elastane core-spun yarn to shrink the produced double-layer weft-knitted fabric [115]. They found puncture failure of this double-layer fabric to be accompanied by fiber cutting and stretching. However, impregnation with epoxy resin increased the maximum load during quasi-static stab tests by more than one order of magnitude, showing that the pure knitted fabric is not really suitable for stab resistance.

**Figure 13:**
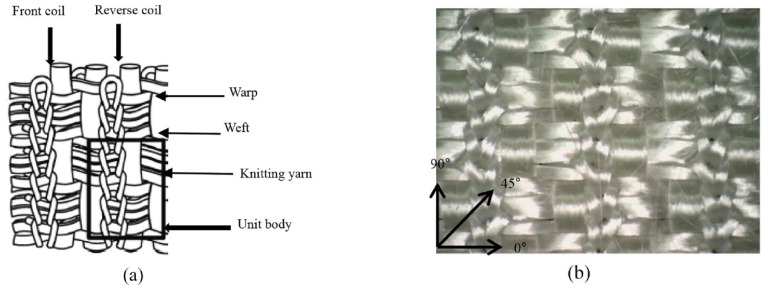
Co-woven-knitted fabric: (**a**) structure diagram, (**b**) fabric map. Reprinted from [116], Copyright (2022) by the authors, originally published under a CC-BY license.

As an effort to reduce the stretchability of knitted fabrics, Zhang et al. prepared co-woven knitted fabrics, as depicted in Figure 13, with E-glass yarn as warp and weft and a polyester knitting yarn [116]. In this way, they found maximum penetration forces around 200 N, which is much higher than values found for pure knitted fabrics [115].

Most often, woven fabrics are used for stab protection, often using aramid or aramid hybrid yarns. Tien et al. prepared aramid-core spun yarns from aramid filaments in the core and cotton staples wrapping them, using a ring-spinning machine [117]. Two strands were plied to avoid snarls in the yarn and corresponding problems during weaving. Plain-woven fabrics of these yarns were investigated in dynamic stab tests according to NIJ standard 0115.00, testing for level 1 with an impact energy of 24 J. The penetration depths were lowest for the densest weaves. The allowed penetration depth of max. 7 mm was reached by 17 layers of the core-spun yarns or more than 60 layers for pure aramid yarns, clearly showing the advantage of the core-spun yarns. The same authors later investigated more hybrid yarns and found basalt/cotton woven fabrics to have the optimum stab-resistant properties [118].

Basalt was also investigated by Li et al., here in the form of a needle-punched, laminated composite with low-melting point PET [119]. The authors found a significant impact of the punching density and the needle punch depth as well as the areal weight of the fabrics.

Fibrilized aramid yarn was the base for plain-weave fabrics investigated by Nasser et al. [120]. The authors found significantly increased yarn-yarn friction in the fibrilized aramid, as compared to the native material, and peak loads six times higher than in the original aramid material, which they attributed to the mechanical interlocking between the fibrilized fibers.

Besides the yarn, the fabric construction method strongly influences the stab resistance of a textile fabric. A typical fabric type is 3D warp interlock fabrics, which combine woven layers with through-the-thickness interlock structures and thus form a 3D structure of a certain thickness. Li et al. compared different 3D warp interlock structures from UHMWPE yarns and found a strong dependence on the structure as well as on the number of layers and their respective orientation [121]. Such fabrics were also investigated in pre-deformed shapes, especially aiming at modeling molded armor panels, as they are used in women’s body armor, where the authors found a significant impact of the stab localization on the fabric [122]. Comparing different orientations of the impinging blade with respect to warp and weft threads, the authors also investigated the depth of trauma in addition to the usual depth of penetration and showed again the strong influence not only of the number of layers, but also of the chosen fabric construction on the depth of penetration as well as the depth of trauma [123].

Another interesting possibility for preparing stab-resistant fabrics is given by triaxial woven fabrics, i.e., fabrics woven from three sets of yarns, oriented approx. 60° with respect to each other. While they are usually not as dense as conventional woven fabrics, the interyarn friction can be increased, thus supporting energy dissipation in such fabrics. Stab resistance of triaxial woven fabrics was described by El Messiry and Eltahan who tested different materials and compared these fabrics with conventional plain weave as well as weft-knitted single jersey fabrics, applying the drop tower test according to NIJ standard 0115.00 [124]. They found the best stab resistance for Vectran triaxial fabrics and only slightly lower values for Kevlar triaxial samples, while the knitted fabrics and even the plain-weave fabrics had significantly lower stab protection. Recently, El Messiry and El-Tarfawy combined triaxial fabrics with weft-knitted fabrics and suggested knitted/triaxial Kevlar multilayer fabrics as optimum regarding the cutting force, suggesting such fabrics also for stab resistance tests [125].

## 10. Conclusions and Outlook

As this review shows, many materials and structures can be used to develop polymer-based stab resistant body armor further. One approach is based on developing new, often bio-inspired structures which can, for example, be produced by different 3D printing techniques. Besides such special structures, most polymer-based stab-resistant garments are based on textile fabrics, either solely or, in most cases, with an additional lamination or coating. Alternatively, composites with embedded fibers or textile fabrics can be prepared, which are stiffer than pure or coated fabrics, but more lightweight than metallic body armor.

While recent research approaches are very often based on impregnating textile fabrics with shear thickening fluids, many more possibilities exist to improve the stab resistant properties of body armor on the yarn or fabric level, by developing materials, structures, and production processes further. In the authors’ opinion, especially combinations of new materials and new shapes, as they can be produced by 3D printing in combination with textile fibers or fabrics [126], offer further improvements for lightweight, yet efficient body armor. With this paper, we hope to inspire more researchers working in these fields to contribute new ideas and experiments to this interesting field of research.

## Data Availability

No new data were created for this review paper.
